# Direct learning-based deep spiking neural networks: a review

**DOI:** 10.3389/fnins.2023.1209795

**Published:** 2023-06-16

**Authors:** Yufei Guo, Xuhui Huang, Zhe Ma

**Affiliations:** ^1^Intelligent Science & Technology Academy of CASIC, Beijing, China; ^2^Scientific Research Laboratory of Aerospace Intelligent Systems and Technology, Beijing, China

**Keywords:** spiking neural network, brain-inspired computation, direct learning, deep neural network, energy efficiency, spatial-temporal processing

## Abstract

The spiking neural network (SNN), as a promising brain-inspired computational model with binary spike information transmission mechanism, rich spatially-temporal dynamics, and event-driven characteristics, has received extensive attention. However, its intricately discontinuous spike mechanism brings difficulty to the optimization of the deep SNN. Since the surrogate gradient method can greatly mitigate the optimization difficulty and shows great potential in directly training deep SNNs, a variety of direct learning-based deep SNN works have been proposed and achieved satisfying progress in recent years. In this paper, we present a comprehensive survey of these direct learning-based deep SNN works, mainly categorized into accuracy improvement methods, efficiency improvement methods, and temporal dynamics utilization methods. In addition, we also divide these categorizations into finer granularities further to better organize and introduce them. Finally, the challenges and trends that may be faced in future research are prospected.

## 1. Introduction

The Spiking Neural Network (SNN) has been recognized as one of the brain-inspired neural networks due to its bio-mimicry of the brain neurons. It transmits information by firing binary spikes and can process the information in a spatial-temporal manner (Wu et al., 2019a; Wu Y. et al., 2019; Zhang et al., 2020a,b; Fang et al., 2021b). This event-driven and spatial-temporal manner makes the SNN very efficient and good at handling temporal signals, thus receiving a lot of research attention, especially recently.

Despite the energy efficiency and spatial-temporal processing advantages, it is a challenge to train deep SNNs due to the firing process of the SNN is undifferentiable, thus making it impossible to train SNNs via gradient-based optimization methods. At first, many works leverage the spike-timing-dependent plasticity (STDP) approach (Lobov et al., 2020), which is inspired by biology, to update the SNN weights. However, STDP cannot help train large-scale networks yet, thus limiting the practical applications of the SNN. There are two widely used effective pathways to obtain deep SNNs up to now. First, the ANN-SNN conversion approach (Han and Roy, 2020; Li et al., 2021a; Bu et al., 2022, 2023; Li and Zeng, 2022; Liu et al., 2022; Wang Y. et al., 2022) converts a well-trained ANN to an SNN by replacing the activation function from ReLU with spiking activation. It provides a fast way to obtain an SNN. However, it is limited in the rate-coding scheme and ignores the rich temporal dynamic behaviors of SNNs. Second, the surrogate gradient (SG)-based direct learning approach (Wu Y. et al., 2018; Fang et al., 2021a; Li et al., 2021b; Guo et al., 2022a) tries to find an alternative differentiable surrogate function to replace the undifferentiable firing activity when doing back-propagation of the spiking neurons. Since SG can handle temporal data and provide decent performance with few time-steps on the large-scale dataset, it has received more attention recently.

Considering the sufficient advantages and rapid development of the direct learning-based deep SNN, a comprehensive and systematic survey on this kind of work is essential. Previously related surveys (Ponulak and Kasinski, 2011; Roy et al., 2019; Tavanaei et al., 2019; Wang et al., 2020; Yamazaki et al., 2022; Zhang D. et al., 2022) have begun to classify existing works mainly based on the key components of SNNs: biological neurons, encoding methods, SNN structures, SNN learning mechanisms, software and hardware frameworks, datasets, and applications. Though such classification is intuitive to general readers, it is difficult for them to grasp the challenges and the landmark work involved. While in this survey, we provide a new perspective to summarize these related works, i.e., starting from analyzing the characteristics and difficulties of the SNN, and then classify them into (i) accuracy improvement methods, (ii) efficiency improvement methods, and (iii) temporal dynamics utilization methods, based on the solutions for corresponding problems or the utilization of SNNs' advantages.

Further, these categories are divided into finer granularities: (i) accuracy improvement methods are subdivided as improving representative capabilities and relieving training difficulties; (ii) efficiency improvement methods are subdivided as network compression techniques and sparse SNNs; (iii) temporal dynamics utilization methods are subdivided as sequential learning and cooperating with neuromorphic cameras. In addition to the classification by using strengths or overcoming weaknesses of SNNs, these recent methods can also be divided into the neuron level, network structure level, and training technique level, according to where these methods actually work. The classifications and main techniques of these methods are listed in Tables 1, 2. Finally, some promising future research directions are provided.

**Table 1:** Overview of direct learning-based deep spiking neural networks: part I.

**Type**		**Method**	**Key technology**	**On the level** ^**⋆**^		
				**NL**	**NSL**	**TTL**
Accuracy improvement	Improving representative capabilities	LSNN (Bellec et al., 2018)	Adaptive threshold	✓		
			LTMD (Wang S. et al., 2022)	Adaptive threshold	✓		
			BDETT (Ding et al., 2022)	Dynamic threshold	✓		
			PLIF (Fang et al., 2021b)	Learnable leak constant	✓		
			Plastic synaptic delays (Yu et al., 2022a)	Learnable leak constant	✓		
			Diet-SNN (Rathi and Roy, 2020)	Learnable leak constant& threshold	✓		
			DS-ResNet (Feng et al., 2022)	Multi-firing & Act before Add-ResNet	✓	✓	
			SNN-MLP (Li W. et al., 2022)	Group LIF	✓		
			GLIF Yao et al., 2022	Unified gated LIF	✓		
			Augmented spikes (Yu et al., 2022b)	Augmented spikes	✓		
			InfLoR-SNN (Shen et al., 2023)	Leaky integrate and fire or burst	✓		
			MT-SNN (Wang et al., 2023)	Multiple threshold approach	✓		
			SEW-ResNet (Fang et al., 2021a)	Act before ADD form-based ResNet		✓	
			MS-ResNet (Hu et al., 2021)	Pre-activation form-based ResNet		✓	
			AutoSNN (Na et al., 2022)	Neural architecture search		✓	
			SNASNet (Kim et al., 2022a)	Neural architecture search		✓	
			TA-SNN (Yao et al., 2021)	Attention mechanism		✓	
			TCJA-SNN (Zhu et al., 2022)	Attention mechanism		✓	
			Real spike (Guo et al., 2022d)	Training-inference decoupled structure		✓	
			IM-loss (Guo et al., 2022a)	Information maximization loss			✓
			RecDis-SNN (Guo et al., 2022c)	Membrane potential distribution loss			✓
			Distilling spikes (Kushawaha et al., 2021)	Knowledge distillation		✓	✓
			Local tandem learning (Yang et al., 2022)	Tandem learning			✓
			sparse-KD (Xu et al., 2023a)	Knowledge distillation			✓
			KDSNN (Xu et al., 2023b)	Knowledge distillation			✓
			SNN distillation (Takuya et al., 2021)	Knowledge distillation			✓
		Relieving training difficulties	SuperSpike (Zenke and Ganguli, 2018)	Fixed surrogate gradient			✓
			LISNN (Cheng et al., 2020)	Fixed surrogate gradient			✓
			IM-Loss (Guo et al., 2022a)	Dynamic surrogate gradient			✓
			Gradual surrogate gradient (Guo et al., 2022a)	Dynamic surrogate gradient			✓
			Differentiable spike (Li et al., 2021b)	Learnable surrogate gradient			✓
			SpikeDHS (Leng et al., 2022)	Differentiable surrogate gradient search			✓
			DSR (Meng et al., 2022)	Differentiation on spike representation			✓
			STDBP (Zhang M. et al., 2022)	Rectified postsynaptic potential function		✓	
			SEW-ResNet (Fang et al., 2021a)	Act before ADD form-based ResNet		✓	
			MS-ResNet (Hu et al., 2021)	Pre-activation form-based ResNet		✓	
			NeuNorm (Wu Y. et al., 2019)	Constructing auxiliary feature maps			✓
			tdBN (Zheng et al., 2021)	Threshold-dependent batch normalization			✓
			BNTT (Kim and Panda, 2021)	Temporal batch normalization through time			✓
			PSP-BN (Ikegawa et al., 2022)	Postsynaptic potential normalization		✓	
			TEBN (Kim and Panda, 2021)	Temporal effective batch normalization			✓
			RecDis-SNN (Guo et al., 2022c)	Membrane potential distribution loss			✓
			TET (Deng et al., 2022)	Temporal regularization loss			✓
			Tandem learning (Wu et al., 2021a)	Tandem learning			✓
			Progressive tandem learning (Wu et al., 2021b)	Progressive tandem learning			✓
			Joint A-SNN (Guo et al., 2023)	Joint training of ANN and SNN			✓

^⋆^NL, neuron Level; NSL, network structure level; TTL, training technique level.

**Table 2:** Overview of direct learning-based deep spiking neural networks: part II.

**Type**		**Method**	**Key technology**	**On the level** ^**⋆**^		
				**NL**	**NSL**	**TTL**
Efficiency improvement	Network compression techniques	Spatio-temporal pruning (Chowdhury et al., 2021)	Spatio-temporal pruning			✓
			SD-SNN (Han et al., 2022)	Pruning-regeneration method			✓
			Grad R (Chen et al., 2021)	Pruning-regeneration method			✓
			Temporal pruning (Chowdhury et al., 2022)	Temporal pruning			✓
			Autosnn (Na et al., 2022)	Neural architecture searching		✓	
			SNASNet (Kim et al., 2022a)	Neural architecture searching		✓	
			Lottery ticket hypothesis (Kim et al., 2022b)	Lottery ticket hypothesis		✓	
			Distilling spikes (Kushawaha et al., 2021)	Knowledge distillation		✓	✓
			Local tandem learning (Yang et al., 2022)	Tandem learning			✓
			sparse-KD (Xu et al., 2023a)	Knowledge distillation			✓
			KDSNN (Xu et al., 2023b)	Knowledge distillation			✓
			SNN distillation (Takuya et al., 2021)	Knowledge distillation			✓
		Sparse SNNs	ASNN (Zambrano and Bohte, 2016)	A lot of adaptive spiking neurons	✓		
				Correlation-based regularization (Han and Lee, 2022)	Correlation-based regularizer			✓
				Superspike (Zenke and Ganguli, 2018)	Heterosynaptic regularization term			✓
				RecDis-SNN (Guo et al., 2022c)	Membrane potential distribution			✓
				Low-activity SNN (Pellegrini et al., 2021)	Regularization term		✓	✓
Temporal dynamics utilization	Sequential learning	Sequence approximation (She et al., 2021)	Dual-search-space optimization			✓
			Sequential learning (Ponghiran and Roy, 2022)	Improved recurrence dynamics	✓		
			SNN_HAR (Li Y. et al., 2022)	Spatio-temporal extraction		✓	
			Robust SNN (Nomura et al., 2022)	Temporal penalty settings		✓	
			Tandem learning-based SNN model (Wu et al., 2020)	Tandem learning			✓
			SG-based SNN model (Bittar and Garner, 2022b)	Surrogate gradient method			✓
			Combination-based SNN (Bittar and Garner, 2022a)	Combination of many techniques	✓	✓	
			Low-activity SNN (Pellegrini et al., 2021)	Regularization term			✓
			SNNCNN (Sadovsky et al., 2023)	Combination of CNNs and SNNs		✓	✓
			RSNNs (Yin et al., 2021)	activity-regularizing SG		✓	✓
	Cooperating with neuromorphic cameras	daptive-spikenet (Kosta and Roy, 2022)	Learnable neuronal dynamics	✓		
		StereoSpike (Rançon et al., 2021)	Modified U-Net-like architecture		✓	✓
		SuperFast (Gao et al., 2022)	Event-enhanced frame interpolation		✓	
		E-SAI (Yu L. et al., 2022)	Synthetic aperture imaging method		✓	
		EVSNN (Zhu L. et al., 2022)	Potential-assisted SNN	✓	✓	
		Spiking-Fer (Barchid et al., 2023)	Deep CSNN		✓	
		Automotive detection (Cordone et al., 2022)	PLIF & SG & Event encoding	✓		✓
		STNet (Zhang J. et al., 2022)	Spiking transformer network		✓	
		LaneSNNs (Viale et al., 2022)	offline supervised learning rule			✓
		HALSIE (Biswas et al., 2022)	Hybrid approach		✓	
		SpikeMS (Parameshwara et al., 2021)	Spatio-temporal loss			✓
		Event-based pose tracking (Zou et al., 2023)	Spiking spatiotemporal transformer		✓	

^*^NL, neuron Level; NSL, network structure level; TTL, training technique level.

The organization of the remaining part is given as follows, Section 2 introduces the preliminary for spiking neural networks. The characteristics and difficulties of the SNN are also analyzed in Section 2. Section 3 presents the recent advances falling into different categories. Section 4 points out future research trends and concludes the review.

## 2. Preliminary

Since the neuron models are not the focus of the paper, here, we briefly introduce the commonly used discretized Leaky Integrate-and-Fire (LIF) spiking neurons to show the basic characteristic and difficulties in SNNs, which can be formulated by


(1) $$\begin{array}{l} U_{l}^{t}=\tau U_{l}^{t-1}+{\text{W}}_{l}O_{l-1}^{t},\;U_{l}^{t}<V_{\text{th}}, \end{array}$$

where $U_{l}^{t}$ is the membrane potential at *t*-th time-step for *l*-th layer, $O_{l-1}^{t}$ is the spike output from the previous layer, **W**_*l*_ is the weight matrix at *l*-th layer, *V*_*th*_ is the firing threshold, and τ is a time leak constant for the membrane potential, which is in (0, 1). When τ is 1, the above equation will degenerate to the Integrate-and-Fire (IF) spiking neuron.

**Characteristic 1**. *Rich spatially-temporal dynamics. Seen from Equation (1), different from ANNs SNNs enjoy the unique spatial-temporal dynamic in the spiking neuron model*.

Then, when the membrane potential exceeds the firing threshold, it will fire a spike and then fall to resting potential, given by


(2) $$O_{l}^{t}=\{\begin{array}{l} 1,\text{ if }U_{l}^{t}\geq V_{\text{th}}\\ 0,\text{ otherwise} \end{array}.$$

**Characteristic 2**. *Efficiency. Since the output is a binary tensor, the multiplications of activations and weights can be replaced by additions, thus enjoying high energy efficiency. Furthermore, when there is no spike output generated, the neuron will keep silent. This event-driven mechanism can further save energy when implemented in neuromorphic hardware*.

**Characteristic 3**. *Limited representative ability. Obviously, transmitting information by quantizing the real-valued membrane potentials into binary output spikes will introduce the quantization error in SNNs, thus causing information loss (Guo et al.*, *2022b**; Wang et al.*, *2023**). Furthermore, the binary spike feature map from a timestep cannot carry enough information like the real-valued one in ANNs (Guo et al.*, *2022d**). These two problems limit the representative ability of SNN to some extent*.

**Characteristic 4**. *Non-differentiability. Another thorny problem in SNNs is the non-differentiability of the firing function*.

To demonstrate this problem, we formulate the gradient at the layer *l* by the chain rule, given by


(3) $$\begin{array}{l} \frac{\partial L}{\partial {\text{W}}_{l}}=\underset{t}{\sum }\left(\frac{\partial L}{\partial O_{l}^{t}}\frac{\partial O_{l}^{t}}{\partial U_{l}^{t}}+\frac{\partial L}{\partial U_{l}^{t+1}}\frac{\partial U_{l}^{t+1}}{\partial U_{l}^{t}}\right)\frac{\partial U_{l}^{t}}{\partial {\text{W}}_{l}}, \end{array}$$

where $\frac{\partial O_{l}^{t}}{\partial U_{l}^{t}}$ is the gradient of firing function at *t*-th time-step for *l*-th layer and is 0 almost everywhere, while infinity at *V*_th_. As a consequence, the gradient descent $\left({\text{W}}_{l}\leftarrow {\text{W}}_{l}-\eta \frac{\partial L}{\partial {\text{W}}_{l}}\right)$ either freezes or updates to infinity.

Most existing direct learning-based SNN works focus on solving difficulties or utilizing the advantages of SNNs. Boosting the representative ability and mitigating the non-differentiability can both improve SNN's accuracy. From this perspective, we organize the recent advances in the SNN field as accuracy improvement methods, efficiency improvement methods, and temporal dynamics utilization methods.

## 3. Recent advances

In recent years, a variety of direct learning-based deep spiking neural networks have been proposed. Most of these methods fall into solving or utilizing the intrinsic disadvantages or advantages of SNNs. Based on this, in the section, we classify these methods into accuracy improvement methods, efficiency improvement methods, and temporal dynamics utilization methods. In addition, these classifications are also organized in different aspects with a comprehensive analysis. Tables 1, 2 summarizes the surveyed SNN methods in different categories.

Note that the direct learning methods can be divided into time-based methods and activation-based methods based on whether the gradient represents spike timing (time-based) or spike scale (activation-based; Zhu Y. et al., 2022). In time-based methods, the gradients represent the direction where the timing of a spike should be moved, i.e., be moved leftward or rightward on the time axis. The SpikeProp (Bohte et al., 2002) and its variants (Booij and tat Nguyen, 2005; Xu et al., 2013; Hong et al., 2019) all belong to this kind of method and they adopt the negative inverse of the time derivative of membrane potential function to approximate the derivative of spike timing to membrane potential. Since most of the time-based methods would restrict each neuron to fire at most once, in Zhou et al. (2021), the spike time is directly taken as the state of a neuron. Thus the relation of neurons can be modeled by the spike time and the SNN can be trained similarly to an ANN. Though the time-based methods enjoy less computation cost than the activation-based methods and many works (Zhang and Li, 2020; Zhu Y. et al., 2022) have greatly improved the accuracy of the field, it is still difficult to train deep time-based SNN models and apply them to large-scale datasets, e.g., ImageNet. Considering the limits of the time-based methods and the topic of summarizing the recent deep SNNs here, we mainly focus on activation-based methods in the paper.

### 3.1. Accuracy improvement methods

As aforementioned, the limited information capacity and the non-differentiability of firing activity of the SNN cause its accuracy loss for wide tasks. Therefore, to mitigate the accuracy loss in the SNN, a great number of methods devoted to improving the representative capabilities and relief training difficulties of SNNs have been proposed and achieved successful improvements in the past few years.

#### 3.1.1. Improving representative capabilities

Two problems result in the representative ability decreasing of the SNN, the process of firing activity will induce information loss, which has been proved in Guo et al. (2022b) and binary spike maps suffer the limited information capacity, which has been proved in Guo et al. (2022d). These problems can be mitigated on the neuron level, network structure level, and training technique level.

##### 3.1.1.1. On the neuron level

A common way to boost the representative capability of the SNN is to make some hyper-parameters in the spiking neuron learnable. In LSNN (Bellec et al., 2018) and LTMD (Wang S. et al., 2022), the adaptive threshold spike neuron was proposed to enhance the computing and learning capabilities of SNNs. Further, a novel bio-inspired dynamic energy-temporal threshold, which can be adjusted dynamically according to input data for SNNs was introduced in the BDETT (Ding et al., 2022). Some works adopted the learnable membrane time constant in spiking neurons (Zimmer et al., 2019; Yin et al., 2020; Fang et al., 2021b; Luo et al., 2022; Yu et al., 2022a). Combining these two manners, Diet-SNN (Rathi and Roy, 2020) simultaneously adopted the learnable membrane leak and firing threshold.

There are also some works focusing on embedding more factors in the spiking neuron to improve its diversity. A multi-level firing (MLF) unit, which contains multiple LIF neurons with different level thresholds thus could generate more quantization spikes with different thresholds was proposed in DS-ResNet (Feng et al., 2022). A full-precision LIF to communicate between patches in Multi-Layer Perceptron (MLP), including horizontal LIF and vertical LIF in different directions was proposed in SNN-MLP (Li W. et al., 2022). SNN-MLP used group LIF to extract better local features. In GLIF (Yao et al., 2022), to enlarge the representation space of spiking neurons, a unified gated leaky integrate-and-fire Neuron was proposed to fuse different bio-features in different neuronal behaviors via embedding gating factors. In augmented spikes (Yu et al., 2022b), a special spiking neuron model was proposed to process augmented spikes, where additional information can be carried from spike strength and latency. This neuron model extends the computation with an additional dimension and thus could be of great significance for the representative ability of the SNN. In LIFB (Shen et al., 2023), a new spiking neuron model called the Leaky Integrate and Fire or Burst was proposed. The neuron model exhibits three modes including resting, regular spike, and burst spike, which significantly enriches the representative capability. Similar to LIFB, MT-SNN (Wang et al., 2023) proposed a multiple threshold approach to firing different spike modes to alleviate the quantization error, such that it could reach a high accuracy at fewer steps.

Different from these works, InfLoR-SNN (Guo et al., 2022b) proposed a membrane potential rectifier (MPR), which can adjust the membrane potential to a new value closer to quantization spikes than itself before firing activity. MPR directly handles the quantization error problem in SNNs, thus improving the representative ability.

##### 3.1.1.2. On the network structure level

**Figure 1:**
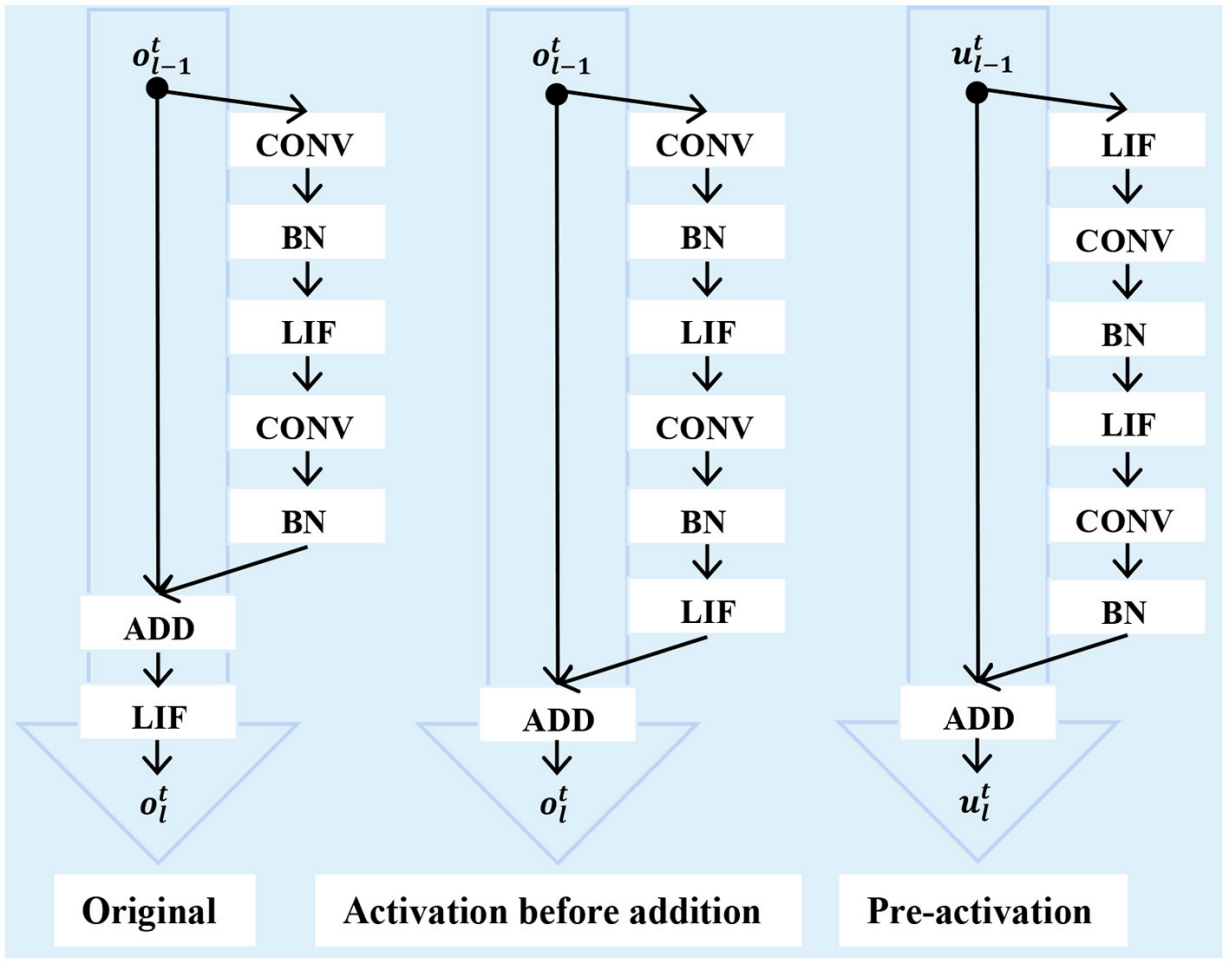
Different SNN ResNet architectures.

To increase the SNN diversity, some works advocate for improving the SNN architecture. In SEW-ResNet (Fang et al., 2021a) and DS-ResNet (Feng et al., 2022), the widely used standard ResNet backbone is replaced by activation before addition form-based ResNet. In this way, the blocks in the network will fire positive integer spikes. Its representation capability will no doubt be increased, however, the advantages of event-driven and multiplication-addition transform in SNNs will be lost in the meantime. To solve the aforementioned problem, MS-ResNet (Hu et al., 2021) adopted the pre-activation form-based ResNet. In this way, the spike-based convolution can be retained. The difference between these methods is shown in Figure 1. However, these SNN architectures are all manually designed. For designing well-performed SNN models automatically, AutoSNN (Na et al., 2022) and SNASNet (Kim et al., 2022a) combined the Neural Architecture Search (NAS) approach to find better SNN architectures. And TA-SNN (Yao et al., 2021) and TCJA-SNN (Zhu et al., 2022) leveraged the learnable attention mechanism to improve the SNN performance.

Different from changing the network topology, Real Spike (Guo et al., 2022d) provides a training-inference decoupled structure. This method enhances the representation capacity of the SNN by learning real-valued spikes during training. While in the inference phase, the rich representation capacity will be transferred from spike neurons to the convolutions by a re-parameterization technique, and meanwhile, the real-valued spikes will be transformed into binary spikes, thus maintaining the event-driven and multiplication-addition transform advantages of SNNs.

Besides, increasing the timestep of SNN will undoubtedly improve the SNN accuracy too, which has been proved in many works (Wu Y. et al., 2018, 2019; Fang et al., 2021a). To some extent, increasing the timestep is equivalent to increasing neuron output bits through the temporal dimension, which will increase the representation capability of feature map (Feng et al., 2022). However, using more timesteps achieves better performance at the cost of increasing inference time.

##### 3.1.1.3. On the training technique level

Some works attempted to improve the representative capability of the SNN on the training technique level, which can be categorized as regularization and distillation. Regularization is a technique that introduces another loss term to explicitly regularize the membrane potential or spike distribution to retain more useful information in the network that could indirectly help train the network as follows,


(4) $$\begin{array}{l} L_{Total}=L_{CE}+\lambda L_{DL} \end{array}$$

where L_*CE*_ is the common cross-entropy loss, L_*DL*_ is the distribution loss for learning the proper membrane potential or spike, and λ is a coefficient to balance the effect of the two types of losses. IM-Loss (Guo et al., 2022a) argues that improving the activation information entropy can reduce the quantization error, and proposed an information maximization loss function that can maximize the activation information entropy. In RecDis-SNN (Guo et al., 2022c), a loss for membrane potential distribution to explicitly penalize three undesired shifts was proposed. Though the work is not designed for reducing quantization error specifically, it still results in a bimodal membrane potential distribution, which has been proven can mitigate the quantization error problem.

The distillation methodology aims to help train a small student model by transferring knowledge of a rather large trained teacher model based on the consensus that the representative ability of a teacher model is better than that of the student model. Recently, some interesting works that introduce the distillation method in the SNN domain were proposed. In Kushawaha et al. (2021), a big teacher SNN model is used to guide the small SNN counterpart learning. While in Yang et al. (2022), Takuya et al. (2021), and Xu et al. (2023a,b) an ANN-teacher is used to guide SNN-student learning. In specific, Local Tandem Learning (Yang et al., 2022) uses the intermediate feature representations of the ANN to supervise the learning of SNN. While in sparse-KD (Xu et al., 2023a), the logit output of the ANN was adopted to guide the learning of the SNN. Furthermore, KDSNN (Xu et al., 2023b) and SNN distillation (Takuya et al., 2021) used both feature-based and logit-based information to distill the SNN.

#### 3.1.2. Relieving training difficulties

The non-differentiability of the firing function impedes the deep SNN direct training. To handle this problem, recently, using the surrogate gradient (SG) function for spiking neurons has received much attention. SG method utilizes a differentiable surrogate function to replace the non-differentiable firing activity to calculate the gradient in the back-propagation (Neftci et al., 2019; Wu Y. et al., 2019; Rathi and Roy, 2020; Fang et al., 2021a). Though the SG method can alleviate the non-differentiability problem, there exists an obvious gradient mismatch between the gradient of the firing function and the surrogate gradient. And the problem easily leads to under-optimized SNNs with severe performance degradation. Intuitively, an elaborately designed surrogate gradient can help to relieve the gradient mismatch in the backward propagation. As a consequence, some works are focusing on designing better surrogate gradients. In addition, the gradient explosion/vanishing problem in SNNs is severer over ANNs, due to the adoption of tanh-like function for most SG methods. There are also some works focusing on handling the gradient explosion/vanishing problem. Note that, these methods in this section can also be classified as the improvement on the neuron level, network structure level, and training technique level, which can be seen in the Table 1. Nevertheless, to better introduce these works, we still organize them as designing the better surrogate gradient and relieving the gradient explosion/vanishing problem.

##### 3.1.2.1. Designing the better surrogate gradient

**Figure 2:**
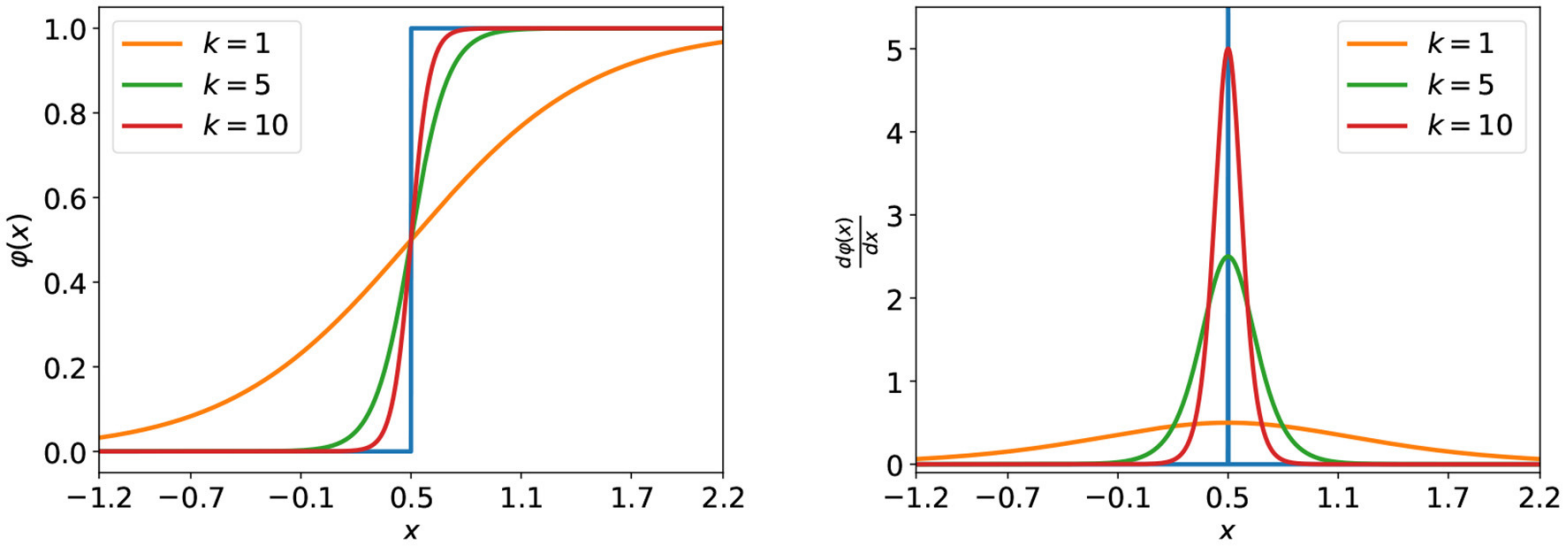
The approximation function **(left)** under different values of the coefficient, *k* and its corresponding gradient **(right)**. The blue curves represent the firing function **(left)** and its true gradient **(right)**.

Most earlier works adopt fixed SG-based methods to handle the non-differentiability problem. For example, the derivative of a truncated quadratic function, the derivatives of a sigmoid, and a rectangular function were respectively adopted in Bohte (2011), Zenke and Ganguli (2018), and Cheng et al. (2020). However, such a strategy would limit the learning capacity of the network. To this end, a dynamic SG method was proposed in Guo et al. (2022a) and Chen et al. (2022), where the SG could change along with epochs as follows,


(5) $$\begin{array}{l} \varphi \left(x\right)=\frac{1}{2}\text{tanh}\left(K\left(i\right)\left(x-V_{\text{th}}\right)\right)+\frac{1}{2} \end{array}$$

where φ(*x*) is the backward approximation function for the firing activity and *K*(*i*) is a dynamic coefficient that changes along with the training epoch as follows,


(6) $$\begin{array}{l} K\left(i\right)=\frac{\left(10^{\frac{i}{N}}-10^{0}\right)K_{max}+\left(10^{1}-10^{\frac{i}{N}}\right)K_{min}}{9} \end{array}$$

where *K*_*min*_ and *K*_*max*_ are the lower bound and the upper bound of *K*, and *i* is the index of epoch starting from 0 to *N*−1. The φ(*x*) and its gradient can be seen in Figure 2. Driven by *K*(*i*), it will gradually evolve to the firing function, thus ensuring sufficient weight updates at the beginning and accurate gradients at the end of the training. Nevertheless, the above SG methods are still designed manually. To find the optimal solution, in Li et al. (2021b), the Differentiable Spike method that can adaptively evolve during training to find the optimal shape and smoothness for gradient estimation based on the finite difference technique was proposed. Then, in Leng et al. (2022), combined with the NAS technique, a differentiable SG search (DGS) method to find the optimized SGs for SNN was proposed. Different from designing a better SG for firing function, DSR (Meng et al., 2022) derived that the spiking dynamics with spiking neural models can be represented as some sub-differentiable mapping and trained the SNNs by the gradients of the mapping, thus avoiding the non-differentiability problem in SNN training.

##### 3.1.2.2. Relieving the gradient explosion/vanishing problem

The gradient explosion or vanishing problem is still severe in SG-only methods. There are three kinds of methods to solve this problem: using improved neurons or architectures, improved batch normalizations, and regularization. In Zhang M. et al. (2022), a simple yet efficient rectified linear postsynaptic potential function (ReL-PSP) for spiking neurons, which benefits for handling the gradient explosion problem, was proposed. On the network architecture level, SEW-ResNet (Fang et al., 2021a) showed that standard spiking ResNet is inapplicable to overcome identity mapping and vanishing/explosion gradient problems and advised using ResNet with activation before addition form. Recently, the pre-activation form-based ResNet was explored in MS-ResNet (Hu et al., 2021). This network topology can simultaneously handle the gradient explosion/vanishing problem and retain the advantages of the SNN.

The normalization approaches are widely used in ANNs to train well-performed models, and these approaches are also introduced in the SNN field to handle the vanishing/explosion gradient problems. For example, NeuNorm (Wu Y. et al., 2019) normalized the data along the channel dimension like BN in ANNs through constructing auxiliary feature maps. Threshold-dependent batch normalization (tdBN; Zheng et al., 2021) considers the SNN normalization from a temporal perspective and extends the scope of BN to the additional temporal dimension. Furthermore, some works (Kim and Panda, 2021; Duan et al., 2022; Ikegawa et al., 2022) argued that the distributions of different timesteps vary wildly, thus bringing a negative impact when using shared parameters. Subsequently, the temporal Batch Normalization Through Time (BNTT), postsynaptic potential normalization (PSP-BN), and temporal effective batch normalization (TEBN) that can regulate the spike flows by utilizing separate sets of BN parameters on different timesteps were proposed. Though adopting temporal BN parameters on different timesteps can obtain more well-performed SNN models, this kind of BN technique can not fold the BN parameters into the weights and will increase the computations and running time in the inference stage, which should also be noticed.

Using the regularization loss can also mitigate the gradient explosion/vanishing problem. In RecDis-SNN (Guo et al., 2022c), a new perspective to further classify the gradient explosion/vanishing difficulty of SNNs into three undesired shifts of the membrane potential distribution was presented. To avoid these undesired shifts, a membrane potential regularization loss was proposed in RecDis-SNN, this loss introduces no additional operations in the SNN inference phase. In TET (Deng et al., 2022), an extra temporal regularization loss to compensate for the loss of momentum in the gradient descent with SG methods was proposed. With this loss, TET can converge into flatter minima with better generalizability.

Since ANNs are fully differentiable to be trained with gradient descent, there is also some work utilizing ANN to guide the SNN's optimization (Wu et al., 2021a,b; Guo et al., 2023). In Wu et al. (2021a) a tandem learning framework was proposed, that consists of an SNN and an ANN that share the same weight. In this framework, the spike count as the discrete neural representation in the SNN would be presented to the coupled ANN activation function in the forward phase. And in the backward phase, the error back-propagation is performed on the ANN to update the shared weight for both the SNN and the ANN. Furthermore, in Wu et al. (2021b), a progressive tandem learning framework was proposed, that introduces a layer-wise learning method to fine-tune the shared network weights. Considering the difference between the ANN and SNN, Joint A-SNN (Guo et al., 2023) developed a partial weight-sharing regime for the joint training of weight-shared ANN and SNN, that applies the Singular Value Decomposition (SVD) to the weights parameters and keep the same eigenvectors while the separated eigenvalues for the ANN and SNN.

### 3.2. Efficiency improvement methods

An important reason why have SNNs received extensive attention recently is that they are seen as more energy efficient than ANNs due to their event-driven computation mechanism and the replacement of energy-consuming weight multiplication with addition. To further explore the efficiency advantages of SNNs so that they can be applied to energy-constrained devices is also a hot topic in the SNN field. This kind of method can be mainly categorized into network compression techniques and sparse SNNs.

#### 3.2.1. Network compression techniques

Network compression techniques have been widely used in ANNs. There are also some works applying these techniques in SNNs. In the literature, approaches for compressing deep SNNs can be classified into three categories: parameter pruning, NAS, and knowledge distillation.

##### 3.2.1.1. Parameter pruning

Parameter pruning mainly focuses on eliminating the redundant parameters in the model by removing the uncritical ones. SNNs, unlike their non-spiking counterparts, consist of a temporal dimension. Along with considering temporal information, a spatial and temporal pruning of SNNs is proposed in Chowdhury et al. (2021). Generally speaking, pruning will cause accuracy degradation to some extent. To avoid this, SD-SNN (Han et al., 2022) and Grad R (Chen et al., 2021) proposed the pruning-regeneration method for removing the redundancy in SNNs from the brain development plasticity mechanism. With synaptic regeneration, these works can effectively prevent and repair over-pruning. Recently, an interesting temporal pruning, which is specific for SNNs, was proposed in Chowdhury et al. (2022). This method starts with an SNN of *T* timesteps and reduces *T* every iteration of training, which results in a continuum of accurate and efficient SNNs from *T* timesteps, down to 1 timestep.

##### 3.2.1.2. Neural architecture searching

Obviously, a compact network carefully designed can reduce the storage and computation complexity of SNNs. However, due to the limitations of humans' inherent knowledge, it is difficult for people to jump out of their original thinking paradigm and design an optimal compact model. Therefore, there are some works using NAS techniques to let the algorithm automatically design the compact neural architecture (Kim et al., 2022a; Na et al., 2022). Furthermore, in Kim et al. (2022b), the lottery ticket hypothesis was investigated which shows that dense SNN networks contain smaller SNN subnetworks, i.e., winning tickets, which can achieve comparable performance to the dense ones, and the smaller compact one is picked as to be used network.

##### 3.2.1.3. Knowledge distillation

The knowledge distillation methods aim at obtaining a compact model from a large model. In Kushawaha et al. (2021), a larger teacher SNN model is used to distill a smaller SNN model. And in Yang et al. (2022), Takuya et al. (2021), and Xu et al. (2023a,b), the same architecture ANN-teacher is used to distill SNN-student.

#### 3.2.2. Sparse SNNs

Different from ANNs, SNNs transmit information by spike events, and the computation occurs only when the neuron receives spike events. Benefitting from this event-driven computation mechanism, SNNs can greatly save energy and run efficiently when implemented on neuromorphic hardware. Hence, limiting the firing rate of spiking neurons to achieve a sparse SNN is also a widely used way to improve the efficiency of the SNN. These kinds of methods can limit the firing rate of the SNN on both the neuron level and training technique level.

##### 3.2.2.1. On the neuron level

In ASNN (Zambrano and Bohte, 2016), an adaptive SNN based on a group of adaptive spiking neurons was proposed. These adaptive spiking neurons can optimize their firing rate using asynchronous pulsed Sigma-Delta coding efficiently.

##### 3.2.2.2. On the training technique level

In Han and Lee (2022), a correlation-based regularizer, which is incorporated into a loss function, was proposed to minimize the redundancies between the features at each layer for structural sparsity. Obviously, this method is beneficial for energy-efficient. Superspike (Zenke and Ganguli, 2018) added a heterosynaptic regularization term to the learning rule of the hidden layer weights to avoid pathologically high firing rates. RecDis-SNN (Guo et al., 2022c) incorporated a membrane potential loss into the SNN to regulate the membrane potential distribution to an appropriate range to avoid high firing rates. In Pellegrini et al. (2021), to enforce sparse spiking activity, a *l*_1_ or *l*_2_ regularization on the total number of spikes emitted by each layer was applied.

### 3.3. Temporal dynamics utilization methods

Different from ANNs, SNNs enjoy rich temporal dynamics characteristics, which makes them more suitable for some particular temporal tasks and some vision sensors with high resolution in time, e.g., neuromorphic cameras, which can capture temporally rich information asynchronously inspired by the information process form of eyes. Given such characteristics, a great number of methods falling in sequential learning and cooperating with neuromorphic cameras have been proposed for SNNs.

#### 3.3.1. Sequential learning

As aforementioned in Section 2, SNNs maintain a dynamic state in the neuron memory. In Ponghiran and Roy (2022), the usefulness of the inherent recurrence dynamics of the SNN for sequential learning was demonstrated, that it can retain important information. Thus, SNNs show better performance on sequential learning compared to ANNs with similar scales in many works. In She et al. (2021), a function approximation theoretical basis was developed that any spike-sequence-to-spike-sequence mapping functions can be approximated by an SNN with one neuron per layer using skip-layer connections. And then, based on the basis, a suitable SNN model for the classification of spatio-temporal data was designed. In Li Y. et al. (2022), SNNs were leveraged to study the Human Activity Recognition (HAR) task. Since SNNs allow spatio-temporal extraction of features and enjoy low-power computation with binary spikes, they can reduce up to 94% energy consumption while achieving better accuracy compared with homogeneous ANN counterparts. In Nomura et al. (2022), an interesting phenomenon was found that SNNs trained with the appropriate temporal penalty settings are more robust against adversarial images than ANNs.

As the common sequential signal, many preliminary works on speech recognition systems based on spiking neural networks have been explored (Tavanaei and Maida, 2017a,b; Wu et al., 2018a,b, 2019b, 2020; Zhang et al., 2019; Hao et al., 2020). In Wu et al. (2020), a deep spiking neural network was trained by the tandem learning method to handle the large vocabulary automatic speech recognition task. The experimental results demonstrated that the deep SNN trained could compete with its ANN counterpart while requiring as low as 0.68 times total synaptic operations to their ANN counterparts. There are also some works training deep SNN directly with SG methods for the speech task. In Ponghiran and Roy (2022), inspired by the LSTM, a custom version of SNNs was defined that combines a forget gate with multi-bit outputs instead of binary spikes, yielding better accuracy than that of LSTMs, but with 2 × fewer parameters. In Bittar and Garner (2022b), the spiking neural networks trained like recurrent neural networks only using the standard surrogate gradient method can achieve promising results on speech recognition tasks, which shows the advantage of SNNs to handle this kind of task. In Bittar and Garner (2022a), a combination of adaptation, recurrence, and surrogate gradient techniques for spiking neural networks was proposed. And with these improvements, light spiking architectures that are not only able to compete with ANN solutions but also retain a high degree of compatibility with them were yielded. In Pellegrini et al. (2021), the dilated convolution spiking layers and a new regularization term to penalize the averaged number of spikes were used to train low-activity supervised convolutional spiking neural networks. The results showed that the SNN models can reach an error rate very close to standard DNNs while very energy efficient for speech tasks. In Sadovsky et al. (2023), a new technique for speech recognition that combines convolutional neural networks with spiking neural networks was presented to create an SNNCNN model. The results showed that the combination of CNNs and SNNs outperforms both MLPs and ANNs, providing a new route to further improvements in the field. In Yin et al. (2021), an activity-regularizing surrogate gradient method combined with recurrent networks of tunable and adaptive spiking neurons for SNNs was proposed, and the method performed well on the speech recognition task.

#### 3.3.2. Cooperating with neuromorphic cameras

Neuromorphic camera, which is also called event-based cameras, have recently shown great potential for high-speed motion estimation owing to their ability to capture temporally rich information asynchronously. SNNs, with their spatio-temporal and event-driven processing mechanisms, are very suitable for handling such asynchronous data. Many excellent works combine SNNs and neuromorphic cameras to solve real-world large-scale problems. In Hagenaars et al. (2021) and Kosta and Roy (2022), an event-based optical flow estimation method was presented. In StereoSpike (Rançon et al., 2021) a depth estimation method was provided. SuperFast (Gao et al., 2022) leveraged an SNN and an event camera to present an event-enhanced high-speed video frame interpolation method. SuperFast can generate a very high frame rate (up to 5,000 FPS) video from the input low frame rate (25 FPS) video. Furthermore, Based on a hybrid network composed of SNNs and ANNs, E-SAI (Yu L. et al., 2022) provided a novel synthetic aperture imaging method, which can see through dense occlusions and extreme lighting conditions from event data. And in EVSNN (Zhu L. et al., 2022) a novel Event-based Video reconstruction framework was proposed. To fully use the information from different modalities, HALSIE (Biswas et al., 2022) proposed a hybrid approach for semantic segmentation comprised of dual encoders with an SNN branch to provide rich temporal cues from asynchronous events, and an ANN branch for extracting spatial information from regular frame data by simultaneously leveraging image and event modalities.

There are also some works that apply this technique in autonomous driving. In Cordone et al. (2022), fast and efficient automotive object detection with spiking neural networks on automotive event data was proposed. In Zhang J. et al. (2022), a spiking transformer network, STNet, which can dynamically extract and fuse information from both temporal and spatial domains was proposed for single object tracking using event data. Besides, since event cameras enjoy extremely low latency and high dynamic range, they can also be used to handle the harsh environment, i.e., extreme lighting conditions or dense occlusions. LaneSNNs (Viale et al., 2022) presented an SNN-based approach for detecting the lanes marked on the streets using the event-based camera input. The experimental results show a very low power consumption of about 1 W, which can significantly increase the lifetime and autonomy of battery-driven systems.

Based on the event-based cameras and SNNs, some works attempted to assist the behavioral recognition research. For examples, Spiking-Fer (Barchid et al., 2023) proposed a new end-to-end deep convolutional SNN method to predict facial expression. SpikeMS (Parameshwara et al., 2021) proposed a deep encoder-decoder SNN architecture and a novel spatio-temporal loss for motion segmentation using the event-based DVS camera as input. In Zou et al. (2023), a dedicated end-to-end sparse deep SNN consisting of the Spike-Element-Wise (SEW) ResNet and a novel Spiking Spatiotemporal Transformer was proposed for event-based pose tracking. This method achieves a significant computation reduction of 80% in FLOPS, demonstrating the superior advantage of SNN in this kind of task.

## 4. Future trends and conclusions

The spiking neural networks, born in mimicking the information process of brain neurons, enjoy many specific characteristics and show great potential in many tasks, but meanwhile suffer from many weaknesses. As a consequence, a number of direct learning-based deep SNN solutions for handling these disadvantages or utilizing the advantages of SNNs have been proposed recently. As we summarized in this survey, these methods can be roughly categorized into (i) accuracy improvement methods, (ii) efficiency improvement methods, and (iii) temporal dynamics utilization methods. Though successful milestones and progress have been achieved through these works, there are still many challenges in the field.

On the accuracy improvement aspect, the SNN still faces serious performance loss, especially for the large network and datasets. The main reasons might include:

- *Lack of measurement of information capacity:* it is still unclear how to precisely calculate the information capacity of the spike maps and what kind of neuron types or network topology is suitable for preserving information while the information passing through the network, even after firing function. We believe SNN neurons and architectures should not be referenced from brains or ANNs completely. Specific designs in regard to the characteristic of SNNs for preserving information should be explored. For instance, to increase the spiking neuron representative ability, the binary spike {0, 1}, which is used to mimic the activation or silence in the brain, can be replaced by ternary spike {-1, 0, 1}, thus the information capacity of the spiking neuron will be boosted, but the event-driven and multiplication-free operation advantages of the binary spike can be preserved still. And as aforementioned, the widely used standard ResNet backbone in ANNs is not suitable for SNNs. And the PreAct ResNet backbone performs better since the membrane potential in neurons before the firing function will be added to the next block, thus the complete information will be transmitted simultaneously. While for the standard ResNet backbone, only quantized information is transmitted. To further preserve the information, adding the shortcut layer by layer in the PreAct ResNet backbone is better in our experiment, which is much different from the architectures in ANNs and is a promising exploration direction.
- *Inherent optimization difficulties:* It is still a difficult problem to optimize the SNN in a discrete space, even though many novel gradient estimators or approximate functions have been proposed, there are still some huge obstacles in the field. Such as the gradient explosion/vanishing problem, with the increasing timestep, the problem along with the gradient errors will become severer and make the network hard to converge. Thus, how to completely eliminate the impact of this problem to directly train an SNN with large timesteps is still under exploration. We believe more theoretical studies and practical tricks will emerge to answer this question in the future.

It is also worth noting that accuracy is not the only criterion of SNNs, the versatility is another key criterion, that measures whether a method can be used in practice. Some methods proposed in prior works are very versatile, such as learnable spike factors proposed in Real Spike (Guo et al., 2022d), membrane potential rectifier proposed in InfLoR-SNN (Guo et al., 2022b), temporal regularization loss proposed in TET (Deng et al., 2022), etc. These methods enjoy simple implementation and low coupling, thus having become common widely used practices to improve the accuracy of SNNs. Some methods improve the accuracy of SNNs by designing complex spiking neurons or specific architectures. Such improvements usually show a stronger ability to increase performance. However, as we have pointed out before, some of them suffer complicated computation and even lose the energy-efficiency advantage, which violates the original intention of SNNs. Therefore, purely pursuing high accuracy without considering versatility has limited significance in practice. The balance between accuracy and versatility is also an essential criterion for SNN research that should be considered in the following works.

On the efficiency improvement aspect, some prior works ignore the important fact, that the event-driven paradigm and friendly to the neuromorphic hardware make SNNs much different from ANNs. When implemented on the neuromorphic hardware, the computation in the SNN occurs only if the spiking neuron receives spike events. Hence, the direct reason for improving the efficiency of the SNN is reducing the the number of the firing spikes, not reducing network size. Some methods intending to improve the efficiency of SNNs by pruning inactive neurons as doing in ANNs can not make sense. We even think that under the condition the SNN network size does not exceed the capacity of the neuromorphic hardware, enlarging the network size but limiting the number of the firing spikes at the same time may be a potential route to improve the accuracy and efficiency simultaneously. In this way, different weights of the SNN may respond to different data, thus being equivalent to improving the representative capabilities of the SNN. However, a more systematic study needs to be done in the future.

On the temporal dynamics utilization aspect, a great number of interesting methods have been proposed and shown wide success. We think it is a very potential direction in the SNN field. Some explainable machine learning-related study indicates that different network types follow different patterns and enjoy different advantages. In this sense, it might be more meaningful to dive into the temporal dynamics of the SNN deeply, but not to pursue higher accuracy as ANNs. Meanwhile, considering the respective advantages, to use ANNs and SNNs together needs to be studied further.

Last but not least, more special applications for SNNs also should be explored still. Though SNNs have been used widely in many fields, including the neuromorphic camera, HAR task, speech recognition, autonomous driving, etc., as aforementioned and the object detection (Kim et al., 2020; Zhou et al., 2020), object tracking (Luo et al., 2020), image segmentation (Patel et al., 2021), robotic (Stagsted et al., 2020; Dupeyroux et al., 2021), etc., where some remarkable studies have applied SNNs on recently, compared to ANNs, their real-world applications are still very limited. Considering the unique advantage, efficiency of SNNs, we think there is a great opportunity for applying SNNs in the Green Artificial Intelligence (GAI), which has become an important subfield of Artificial Intelligence and has notable practical value. We believe many studies focusing on using SNNs for GAI will emerge soon.

## Author contributions

YG and XH wrote the paper with ZM being active contributors toward editing and revising the paper as well as supervising the project. All authors contributed to the article and approved the submitted version.
